# Modeling long-term tumor growth and kill after combinations of radiation and radiosensitizing agents

**DOI:** 10.1007/s00280-019-03829-y

**Published:** 2019-04-11

**Authors:** Tim Cardilin, Joachim Almquist, Mats Jirstrand, Astrid Zimmermann, Floriane Lignet, Samer El Bawab, Johan Gabrielsson

**Affiliations:** 1 Fraunhofer-Chalmers Centre, Chalmers Science Park, Gothenburg, Sweden; 2 Department of Mathematical Sciences, Chalmers University of Technology and University of Gothenburg, Gothenburg, Sweden; 3 Translation Innovation Platform Oncology, Merck Healthcare KGaA, Darmstadt, Germany; 4 Translational Medicine, Quantitative Pharmacology, Merck Healthcare KGaA, Darmstadt, Germany; 5 Department of Biomedical Sciences and Veterinary Public Health, Swedish University of Agricultural Sciences, Uppsala, Sweden

**Keywords:** PK/PD, Oncology, Radiation therapy, Combination therapy, Turnover model, Interspecies scaling, Translational science

## Abstract

**Purpose:**

Radiation therapy, whether given alone or in combination with chemical agents, is one of the cornerstones of oncology. We develop a quantitative model that describes tumor growth during and after treatment with radiation and radiosensitizing agents. The model also describes long-term treatment effects including tumor regrowth and eradication.

**Methods:**

We challenge the model with data from a xenograft study using a clinically relevant administration schedule and use a mixed-effects approach for model-fitting. We use the calibrated model to predict exposure combinations that result in tumor eradication using Tumor Static Exposure (TSE).

**Results:**

The model is able to adequately describe data from all treatment groups, with the parameter estimates taking biologically reasonable values. Using TSE, we predict the total radiation dose necessary for tumor eradication to be 110 Gy, which is reduced to 80 or 30 Gy with co-administration of 25 or 100 mg kg^−1^ of a radiosensitizer. TSE is also explored via a heat map of different growth and shrinkage rates. Finally, we discuss the translational potential of the model and TSE concept to humans.

**Conclusions:**

The new model is capable of describing different tumor dynamics including tumor eradication and tumor regrowth with different rates, and can be calibrated using data from standard xenograft experiments. TSE and related concepts can be used to predict tumor shrinkage and eradication, and have the potential to guide new experiments and support translations from animals to humans.

## Introduction

Radiation therapy is one of the leading treatment modalities in modern oncology, with a utilization rate of about 50% [1]. Treatments with ionizing radiation aim to destroy cancerous cells while limiting the damage to the surrounding tissues [2]. The primary mode of cell killing is through induced single- and double-strand breaks in DNA that, if not repaired, result in cell death through mechanisms such as apoptosis and mitotic catastrophe [3]. Successful treatment is contingent on accurate delivery and on host cells exhibiting superior repair mechanisms compared to their cancerous counterparts [2]. Biological tumor features with established impact on treatment outcome include hypoxia, ability to repopulate, and inherent radioresistance. The identification of such features has facilitated the development of targeted molecules that sensitize cancer cells to radiation or protect the surrounding tissue [4]. Modulating the response to DNA damage, e.g., through prevention of non-homologous end-joining and homologous recombination, the main repair mechanisms of double-strand breaks as well as single-strand break repair mechanisms such as base excision repair, have emerged as popular treatment strategies [4]. Moreover, recent successes of immunotherapeutic treatments in advanced cancers have paved the way for combinations of immunotherapy with radiation [5]. There is also evidence to suggest that ionizing radiation can act as an immune modulator and enhance immune recognition of cancerous tumors, e.g., through the release of tumor antigens from dying cells [5].

Integration of quantitative techniques to support efficient study designs and dose selections plays an increasingly important role in pharmaceutical development, including oncology [6]. Performing experiments in silico can also lead to faster, cheaper, and more ethical drug development by decreasing the number of in vivo experiments [7]. Semi-mechanistic models of chemical interventions are regularly employed in preclinical oncology to make predictions based on volume–time data collected from xenograft studies [6, 8]. Applications also include assessing drug synergies and comparing different treatments [9, 10]. For radiation therapy, the de facto means of calculating cell survival is given by the linear-quadratic (LQ) model, which describes the probability of cell survival using a linear and a quadratic term in dose [11]. The LQ model has multiple mechanistic interpretations, e.g., relating the quadratic term to binary misrepair of double-strand breaks produced by different radiation tracks (i.e., different particles) and the linear term to lethal lesions produced by one radiation track [12]. The LQ model is proven to yield accurate predictions for dose fractions up to 18 Gy, and contains sufficiently few parameters to be practically useful [12]. Quantitative models at different scales have been proposed to describe tumor dynamics after radiation therapy [11]. Two simple models featured by Sachs et al. and Schättler and Ledzewics shared the common feature of capturing the LQ prediction of the surviving cell fraction [13, 14]. In a previous analysis, Cardilin et al. proposed a semi-mechanistic model for combinations of ionizing radiation and radiosensitizing treatment that agrees with the LQ prediction [15]. However, the model does not account for long-term effects such as tumor eradication and regrowth dynamics.

**Fig. 1:**
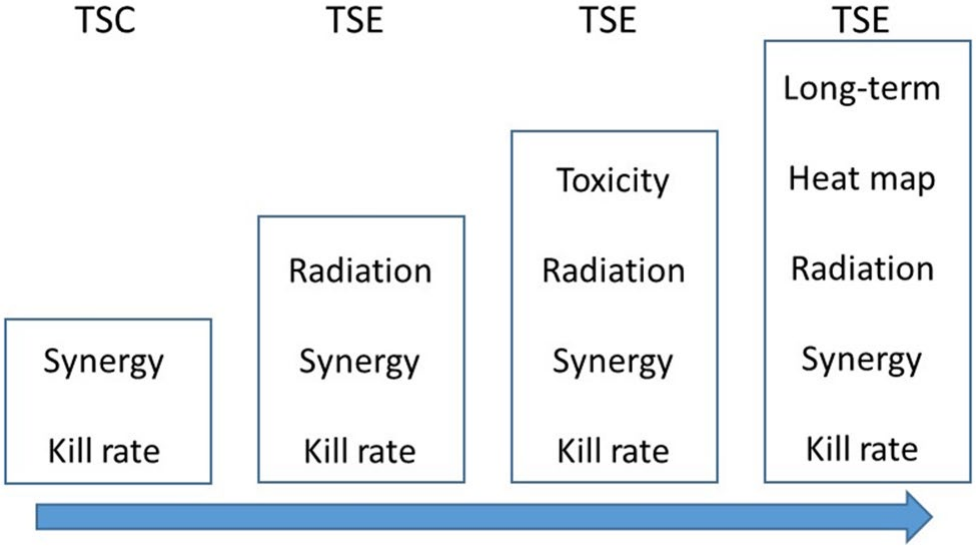
Schematic illustration of development of tumor models and TSE concepts

The analysis presented here is the fourth in a series of quantitative approaches to tumor growth data. A schematic illustration of the progression of tumor models and Tumor Static Exposure (TSE) concepts through these analyses is shown in Fig. 1. The first paper proposed a tumor model for combinations of the anticancer drugs cetuximab and cisplatin [16]. One important feature was the inclusion of a natural death rate of cancer cells. The main contribution was the introduction of the Tumor Static Concentration (TSC) concept for combinations of two or more drugs that intervene with tumor volume, and its connection to drug synergies. In particular, a synergistic drug effect leads to a more convex (curving inward) TSC curve, whereas an antagonistic effect results in a more concave (curving outward) TSC curve [16]. A subsequent analysis presented a tumor model for combinations of radiation and chemical provocation that complied with the LQ prediction in radiobiology [15]. Radiation-induced cell killing was described as triggering apoptosis (possibly lumped with other death mechanisms) in lethally irradiated cells. The analysis also featured an extension of the TSC concept to combinations of radiation and chemicals called TSE. A third analysis showed how TSE can be used to rank and compare combinations of drugs (and radiation) by relating the ability to achieve tumor regression, i.e., TSE, to toxicity (Cardilin et al. 2019, preprint). The analysis also demonstrated the applicability of the previous radiation model by applying it to combinations of radiation and three different radiosensitizers. Moreover, the TSE concept was extended to Tumor Shrinkage Exposures (TSE_dV_) that identify drug combinations that result in a particular shrinkage rate. In the current study, we extend the previous model to long-term radiochemical intervention by complementing radiation-induced apoptosis with inhibition of growth that can be linked to changes in the tumor microenvironment, and the repair/misrepair of lethal lesions. The impact of radiosensitizing intervention is described enhancing both radiation effects. The proposed model captures tumor eradication as well as tumor regrowth with different rates. In particular, it allows for tumor regrowth that is slower than for unirradiated tumors. We then challenged the model by data from a xenograft study using a clinically relevant treatment protocol. Three different TSE curves are introduced based on short-term radiochemical effects, long-term radiochemical effects, and a combination of both. Furthermore, TSE is accompanied by a heat map that illustrates gradual tumor growth or shrinkage associated with different combinations of radiation and radiosensitizer. Finally, we discuss the translational potential of the model and the TSE curve, particularly through an allometric scaling approach.

## Methods

### Experimental data

Tumor volume data were generated in FaDu xenograft mice models. Five-to-six-week-old female CD1 (nu/nu) or NMRI (nu/nu) mice were used (Charles River Laboratories, Sulzfeld, Germany). The animals were kept in groups of ten in polysulfone cages (26.5 × 20.5 × 14 cm) with a room temperature of 24 ± 2 °C and a light cycle of 12 h of light and 12 h of darkness. Drinking water and sterile high- protein maintenance diet were provided ad libitum. Mice received subcutaneous injections in the right lower back [low number of fractions (= 5)] or the right thigh [high number of fractions (= 30)] with 2.5 million FaDu cells (ATCC). When tumor xenografts reached a mean volume of about 50–110 mm^3^, the mice were treated with radiation (local tumor irradiation, X-RAD320 irradiation cabinet Precision X-ray Inc., 15 mA, 250 kV, $58$ s, $50 \;{\text{cm}}$ FSD, collimator, $2 \;{\text{mm}}$ A1 filter) either alone or together with a radiosensitizing agent. Irradiation took place $30$ min after radiosensitizer application. The radiosensitizer is a small-molecule targeted therapy that interferes with the repair of DNA damage. Tumor length ($L$) and width ($W$) were measured with calipers twice a week for up to 12 weeks after treatment arrest. Tumor volumes $(V)$ were calculated using the formula $V = L \times W^{2} /2$. Mice were sacrificed at the end of the experiment, or according to the criteria defined by GV-Solas (Gesellschaft für Versuchstierkunde, Germany). Data were collected in Study Advantage™.

Pharmacodynamic data were based on 40 mice with $N = 10$ in each of the following four groups: vehicle control, radiation treatment (2 Gy), and combination treatment with radiation (2 Gy) and radiosensitizer (25 or 100 mg kg^−1^). Animals received treatment 5 days a week (Mon–Fri) for 6 weeks.

Pharmacokinetic data were based on eight animals. Half ($N = 4$) were given an oral dose of 25 mg kg^−1^ of the radiosensitizer, and the other half ($N = 4$) received an oral dose of 100 mg kg^−1^. Plasma samples were taken 1, 2, and 6 h after dosing. Quantitative determination of plasma concentrations was performed using HPLC–MS/MS assay.

All experiments were approved in accordance with the German animal welfare regulations by the Regierungspräsidium Darmstadt, Hessen, Germany (protocol registration numbers DA 4/Anz. 397 and DA 4/Anz. 398).

### Exposure to radiosensitizer

Exposure to the radiosensitizer was described using a standard one-compartment pharmacokinetic model represented by the following differential equation:

1 $$\frac{{{\text{d}}C}}{{{\text{d}}t}} = - k_{\text{e}} C, C\left( 0 \right) = \frac{DF}{V},$$

where $k_{\text{e}}$ is the elimination rate constant, $V$ the distribution volume, $F$ the bioavailability, $D$ the dose administered at time $t = 0$, and $C$ the plasma concentration of the test compound.

### Tumor model of radiation and radiosensitizer combination treatment

**Fig. 2:**
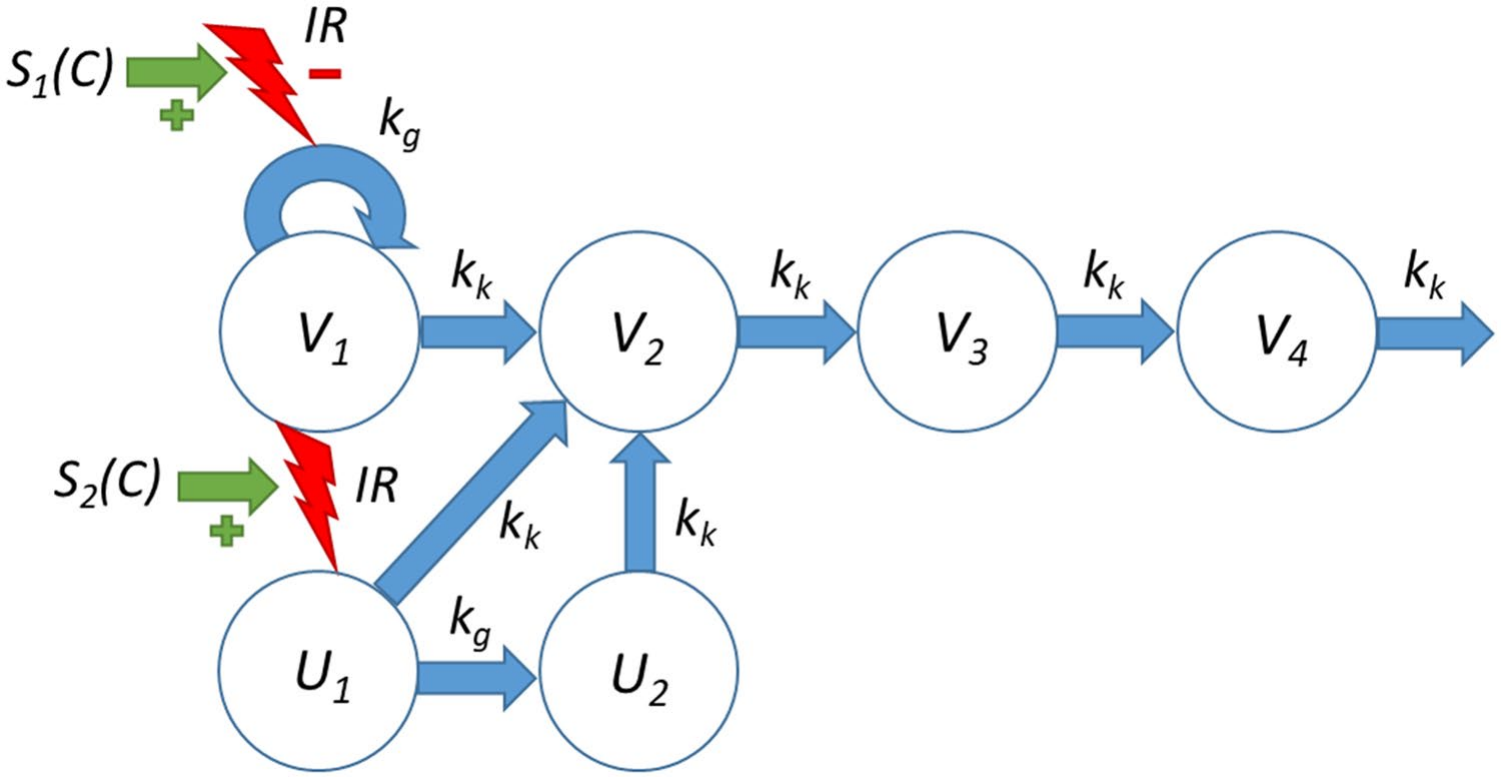
Tumor model for radiation and radiosensitizer combination treatment. Ionizing radiation (IR) induces apoptosis in a fraction of proliferating cancer cells. The model also includes a long-term radiation effect whereby the tumor growth rate is inhibited. $V_{1}$ denotes proliferating cancer cells, *V*_2_, *V*_3_, and *V*_4_ dying cells with different degree of damage, *U*_1_ lethally irradiated cells that are capable of up to one more cell division before starting a series of transitions leading to cell death, *U*_2_ lethally irradiated cells that cannot proliferate, IR the effect of ionizing radiation, *S*_1_ and *S*_2_ the stimulatory effects of radiosensitizing treatment on the short- and long-term radiation effects, respectively, $k_{g}$ the tumor growth rate, and $k_{k}$ a kill rate parameter related to transitions leading to cell death

A tumor model was developed to describe treatment effects of ionizing radiation (IR) and radiosensitizing agents on tumor volume. The model is an extension of an existing model (see [15]) to account for long-term growth dynamics. A schematic illustration of the model is shown in Fig. 2.

The model consists of a main compartment $V_{1}$ of proliferating cancer cells and three transit compartments, $V_{2}$, $V_{3}$, and $V_{4}$, which damaged cells must go through before dying. The model also includes natural cell death, meaning that some cells traverse the transit compartments even for untreated tumors. Two effects of ionizing radiation are described by the model: immediate cell killing of proliferating cells by triggering apoptosis or other death mechanisms, and inhibition of the proliferating capabilities of the surviving cells. Immediate cell killing sends a fraction of the proliferating cells to the compartment $U_{1}$ after which the cells are allowed up to one more cell division (and in the process transferring the daughter cells to the compartment $U_{2}$) before dying via the transit compartments $V_{2}$, $V_{3}$, and $V_{4}$. Such an effect was used in an earlier analysis (see [15]) and is supported by experiments, showing that irradiated cells can survive one or multiple cell cycles before dying through mitotic catastrophe [17, 18]. The second radiation effect describes an inhibition of the growth rate of the surviving cells. This is a regularly observed phenomenon that can be due to mutations and reduced vascularization in the tumor, as well as changes in the tumor microenvironment [19]. The degree of inhibition depends on the accumulated radiation dose, ${\text{IR}}_{\text{Tot}}$, to describe effects that linger beyond the treatment period. Finally, radiosensitizing treatment is described as modulating both radiation effects. More precisely, the radiation effects are enhanced depending on the radiosensitizer exposure at the time of irradiation. Turnover of proliferating cells in $V_{1}$ is described by the equation:

2 $$\begin{aligned} & \frac{{{\text{d}}V_{1} }}{{{\text{d}}t}} = k_{\text{g}} I\left( {{\text{IR}}_{\text{Tot}} } \right)V_{1} - k_{k} V_{1} , \quad t \ne t_{i} \\ & V_{1} (t_{i}^{ + } ) = V_{1} (t_{i}^{ - } ) - F\left( {D_{{t_{i} }} ,C_{{t_{i} }} } \right)V_{1} , \quad i = 1, \ldots , n , \\ \end{aligned}$$

where $k_{\text{g}}$ and $k_{\text{k}}$ are the growth and kill rate, respectively. During irradiation at times $t_{i}$ a fraction $F$ of viable cells will be lethally irradiated and therefore transferred from $V_{1}$ to $U_{1}$. Here, $V_{1} (t_{i}^{ - } )$ and $V_{1} \left( {t_{i}^{ + } } \right)$ denote the volume of cells in $V_{1}$ before and after irradiation at time $t_{i}$, respectively. The lethally irradiated fraction $F$ depends on the radiation dose $D_{{t_{i} }}$ and concurrent radiosensitizer concentration $C_{{t_{i} }}$ and is given by the following equation:

3 $$F\left( {D,C} \right) = 1 - \exp \left[ { - \left( {1 + aC} \right) (\alpha D + \beta D^{2} )} \right],$$

where $\alpha$ and $\beta$ are the linear and quadratic parameters in the LQ model of radiobiology [10], and $a$ is a parameter associated with the potency of the radiosensitizer. Radiation-induced inhibition of growth is given by the function $I$:

4 $$I\left( {{\text{IR}}_{\text{Tot}} } \right) = \exp ( - \gamma {\text{IR}}_{\text{Tot}} ),$$

where $\gamma$ is a radiation parameter associated with the degree of growth inhibition. Turnover of dying cells in the damage compartments $V_{2} , \ldots , V_{4}$ is described by the following equations:

5 $$\begin{aligned} \frac{{{\text{d}}V_{2} }}{{{\text{d}}t}} & = k_{k} V_{1} + k_{k} U_{1} + k_{k} U_{2} - k_{k} V_{2} \\ \frac{{{\text{d}}V_{3} }}{{{\text{d}}t}} & = k_{k} V_{2} - k_{k} V_{3} \\ \frac{{{\text{d}}V_{4} }}{{{\text{d}}t}} & = k_{k} V_{3} - k_{k} V_{4} . \\ \end{aligned}$$

The compartments $U_{1}$ and $U_{2}$ allow lethally irradiated cells up to one more cell division and are governed by the following equations:

6 $$\begin{aligned} & \frac{{{\text{d}}U_{1} }}{{{\text{d}}t}} = - k_{g} U_{1} - k_{k} U_{1} , \quad t \ne t_{i} \\ & U_{1} \left( {t_{i}^{ + } } \right) = U_{1} \left( {t_{i}^{ - } } \right) + F\left( {D_{{t_{i} }} ,C_{{t_{i} }} } \right)V_{1} , \quad i = 1, \ldots , n \\ & \frac{{{\text{d}}U_{2} }}{{{\text{d}}t}} = 2k_{g} U_{1} - k_{k} U_{2} , \\ \end{aligned}$$

with the function $F$ and the parameters $k_{\text{g}}$ and $k_{\text{k}}$ defined as above. Here, $U_{1} (t_{i}^{ - } )$ and $U_{1} \left( {t_{i}^{ + } } \right)$ denote the volume of cells in $U_{1}$ before and after irradiation at time $t_{i}$, respectively. Note that the equation for $U_{1}$ has a term $- k_{\text{g}} U_{1}$, whereas the equation for $U_{2}$ has the term $2k_{\text{g}} U_{1}$. The factor 2 represents that, for every cell that leaves $U_{1}$ (by triggering mitosis), two daughter cells enter $U_{2}$. The accumulated radiation dose ${\text{IR}}_{\text{Tot}}$, after accounting for radiosensitizing enhancement, is given by the following:

7 $${\text{IR}}_{\text{Tot}}(t_i^+) = {\text{IR}}_{\text{Tot}}(t_i^-)+ \left( {1 + bC_{{t_{i} }} } \right)D_{{t_{i} }} ,$$

where $b$ is a potency parameter associated with the radiosensitizer. Here, ${\text{IR}}_{\text{Tot}}(t_i^-)$ and ${\text{IR}}_{\text{Tot}}(t_i^+)$ denote the accumulated radiation dose before and after the *i*th dose at time ${t_i}$. Thus, the accumulated radiation dose is modulated by the radiosensitizer depending on the plasma exposure at the time of irradiation. The initial conditions for all model compartments are given by the following:

8 $$V_{i} \left( 0 \right) = V^{0} \left( {\frac{{k_{\text{k}} }}{{k_{\text{g}} }}} \right)^{i - 1} , \quad U_{j} \left( 0 \right) = 0, \quad {\text{IR}}_{\text{Tot}} \left( 0 \right) = 0.$$

Note that not all initial cells are assumed to be viable. Some of the initial volume will consist of dying cells in the damage compartments $V_{2} , \ldots , V_{4}$, which is consistent with the presence of a natural kill rate. The distribution of initial volume among the compartments is done to ensure strictly exponential growth (see [16]).

### Tumor static exposure

The previous publications have derived the concepts of TSC and TSE, i.e., the exposures of one or multiple compounds that result in tumor stasis [15, 16, 20]. The transition from TSC to TSE was made to include exposure metrics other than plasma concentrations, in particular doses of ionizing radiation [15]. For combinations of radiation and radiosensitizer, the associated TSE curve consists of all pairs of radiation dose and plasma concentration, such that exposures above the curve will lead to tumor regression. One can derive a TSE curve based on the long-term treatment effect using the model in Eq. 2. The TSE curve is derived from the equation for $V_{1}$ by considering for which total radiation doses and radiosensitizer concentrations the growth rate becomes equal to the natural death rate. Any exposure combination above this level will result in a negative net growth rate and, therefore, tumor shrinkage. In these calculations, the short-term radiation effect can be ignored, since it only has a temporary effect on tumor volume. From Eq. 2, the growth and kill rates will be equal when

9 $$k_{\text{net}} \text{ := }k_{\text{g}} \exp ( - \alpha {\text{IR}}_{\text{Tot}} ) - k_{k} = 0,$$

where $k_{net}$ is the net growth rate. Moreover, the (effective) total radiation dose, after an effective increase due to radiosensitizing treatment is accounted for, is given by $\left( {1 + a C} \right)D$, where total radiation dose and radiosensitizer concentration are denoted *D* and $C$, respectively. Inserting this into Eq. 9 yields the following:

10 $$k_{\text{g}} \exp ( - \alpha \left( {1 + a C} \right)D) - k_{\text{k}} = 0.$$

Equation 10 describes a curve in the plane with the plasma concentration $C$ along the horizontal axis, and the total radiation dose and the total radiation dose $D$ along the vertical axis. Equation 10 can be solved for $D$ to obtain the following:

11 $$\frac{{D = \log (k_{\text{g}} /k_{\text{k}} )}}{\alpha (1 + a C)}.$$

Thus, for each value of the plasma exposure of the radiosensitizer $C$, the right-hand side of Eq. 11 gives the necessary total radiation dose $D$, such that the tumor will eventually be eradicated. Equation 11 can be viewed as a function:

12 $$D = f(\theta ;\;C),$$

where $\theta$ is the vector of parameters (here $k_{\text{g}} , \;k_{\text{k}} , \;\alpha , \;{\text{and }}a$). The graph of this function will be the TSE curve. Alternatively, one can solve for $C$ in Eq. 11 to obtain

13 $$C = \frac{{\log (k_{\text{g}} /k_{\text{k}} )}}{a\alpha D} - \frac{1}{a},$$

which describes the same curve as Eq. 11. Similarly, Eq. 13 can be viewed as a function $C = g(\theta ;\;D)$ that, for every radiation dose, $D$ determines the corresponding radiosensitizer concentration $C$, such that the combination will lead to tumor eradication.

### Computational methods

Model-fitting was performed using a mixed-effects approach based on a first-order conditional estimation (FOCE) method in a computational framework developed at the Fraunhofer–Chalmers Research Centre for Industrial Mathematics (Gothenburg, Sweden) and implemented in Mathematica (Wolfram Research) [21]. The tumor model was simultaneously fitted to tumor volume data from all four treatment arms. As in a previous publication, the quotient ${\alpha / \beta}$ was set to the typical value of 10 [15]. Model evaluation was based on individual fit, empirical Bayes estimates (EBEs), residual analysis, and visual predictive checks.

## Results

### Exposure to radiosensitizer

**Fig. 3:**
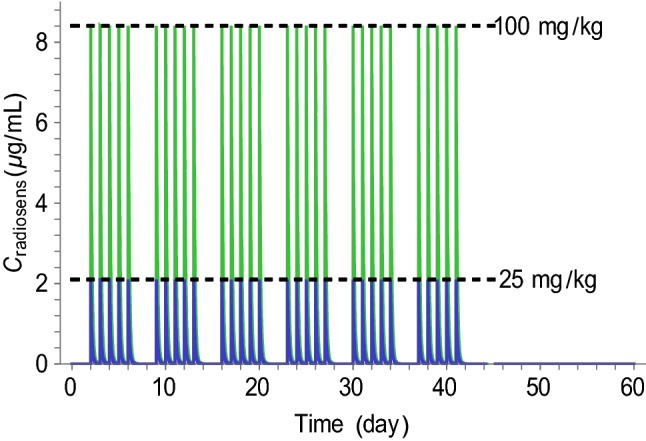
Exposure to the radiosensitizing agent. Treatment was given 5 days a week for 6 weeks. Mice received daily doses of 25 mg kg^−1^ (blue) or 100 mg kg^−1^ (green) via the oral route

Exposure to the radiosensitizer was described using a standard one-compartment pharmacokinetic model. The compound was characterized by a short half-life (3 h), resulting in no accumulation of drug with 24 h dosing intervals. The doses of 25 and 100 mg kg^−1^ lead to peak plasma concentrations of 2 and 8 µg mL^−1^, respectively. Simulated exposure profiles following daily doses of 25 mg kg^−1^ and 100 mg kg^−1^ 5 days a week for 6 weeks are shown in Fig. 3.

### Tumor model of radiation and radiosensitizer combination treatment

The tumor model adequately described the xenograft data from each of the four treatment groups. Table 1 shows the parameter estimates obtained from simultaneously fitting the model to all data using a mixed-effects approach. System and radiation parameters were estimated with good precision (RSE < 20%), whereas drug parameters had RSEs of 31% and 35%. Between-subject variability was considerable (CV >40%) for all three parameters with population variability. There was negligible shrinkage (< 10%) for the parameters $V_{0}$ and $\alpha$ and some shrinkage for $\gamma$ (28%), due to animals receiving radiation and 100 mg kg^−1^ radiosensitizer showing similar growth profiles. Figure 3 shows the individual fit for two mice from each treatment group. Vehicle growth (Fig. 3a, b) was approximately exponential. Radiation treatment led to considerable tumor regression, but led to regrowth in 7/9 mice. In contrast, combination treatment with the radiosensitizer dose of 25 mg kg^−1^ led to tumor eradication in 6/9 mice, and with the higher dose of 100 mg kg^−1^, all tumors were eradicated. A version of Fig. 3 with all data plotted on the same time and volume scales is provided in Appendix A (Fig. 8).

**Table 1:** Pharmacodynamic parameter estimates for the tumor model describing the effects of radiation and radiosensitizer combination treatment

Parameter	Population median (RSE%)	Between-subject variability (RSE%)
$k_{\text{g}}$ (day^−1^)	0.40 (4)	–
$k_{\text{k}}$ (day^−1^)	0.26 (5)	–
$V^{0}$ (mm^3^)	27.0 (7)	50 (9)
$\gamma$ (kGy^−1^)	4.0 (14)	46 (14)
$\alpha$ (kGy^−1^)	54.0 (18)	42 (10)
*a* (mL μg^−1^)	0.42 (31)	–
*b* (mL μg^−1^)	0.15 (35)	–
$\sigma$^a^ (%)	24.0 (3)	–
$\sigma$^b^ (mm^3^)	6.9 (5)	–

^a^Proportional error

^b^Additive error

Using the estimated parameter values, one can derive a tumor doubling time of 5 days for untreated animals. The median initial tumor volume was estimated to 81 mm^3^. Moreover, the estimated value of 0.082 Gy^−1^ for the parameter $\alpha$ corresponds to 15% of proliferating cells dying after each fraction of 2 Gy. Using the estimated value of 0.0034 Gy^−1^ for the long-term radiation effect, the model predicts that a total dose above 120 Gy (i.e., double the current dose) is required for tumor eradication. When radiation was combined with radiosensitizing treatment (25 or 100 mg kg^−1^ per dose), 19% or 25% of proliferating cells, respectively, were killed after each fraction of radiation, and the predicted total radiation dose necessary for tumor eradication was lowered to 80 Gy or 30 Gy, respectively. Visual predictive checks and EBEs for all four treatment arms are shown in Appendix A (Figs. 9 and 10).

Figure 5 shows a simulation of how the net growth rate given by Eq. 9 changes over time for each of the four treatment groups. Radiation-induced inhibition of growth depends of accumulated dose, and hence, the effect is permanent and growth rate will continually decrease with additional radiation doses. Vehicle control (curve A) remains unchanged, whereas radiation alone (curve B) decreases growth rate, but does not result in a negative rate with tumor shrinkage. Combination treatment with 25 mg kg^−1^ of the radiosensitizer (curve C) leads to a net growth rate that barely becomes negative, meaning that this combination is sufficient for tumor eradication for a typical individual. Finally, combination treatment with radiation and 100 mg kg^−1^ of the radiosensitizer and radiation (curve D) leads to a growth rate that is clearly negative.

### Tumor static exposure

The TSE curve for different radiation and radiosensitizer combinations was computed using Eq. 7 together with the parameter estimates from Table 1 and is shown in Fig. 6 (left). The TSE curve shows that a radiation dose of 120 Gy is required for tumor shrinkage, which is reduced to 80 and 30 Gy during co-administration with 25 and 100 mg kg^−1^ of the radiosensitizer, respectively. There is no TSE value corresponding to radiosensitizer treatment alone, due to its lack of intrinsic activity. Figure 6 (right) illustrates tumor growth following exposure below, at, or above the TSE curve, leading to tumor growth, stasis, or eradication, respectively. The associated exposure combinations (A, B, or C) have been marked on the TSE curve.

TSE also varies within the population. One can compute a TSE curve for each individual using the EBEs obtained from mixed-effect modeling (Fig. 7, left). The individual TSE curves are shown in blue, whereas the TSE curve for the median (Fig. 7, left) is shown in red. There is particularly large variability in the total radiation dose required for tumor eradication, with doses ranging from 50 Gy to above 200 Gy across the population. To complement Fig. 7, a sensitivity analysis of the median TSE curve was performed (Appendix A, Fig. 11), which shows that TSE changes the most when either $k_{\text{g}}$ or $k_{\text{k}}$ is adjusted.

Although the TSE curve differentiates between tumor growth and tumor shrinkage, it does not provide information about the growth rate or shrinkage rate associated with a particular combination. Figure 7 (right) shows a heat map of the net tumor growth rate after combinations of radiation and radiosensitizer treatment for the median individual. The different colors represent different growth or shrinkage rates, with the red region in the top right corresponding to shrinkage rates around − 0.2 h^−1^, and the bottom left, to growth rates around 0.1 h^−1^. Each exposure combination corresponds to a specific growth or shrinkage rate. A three-dimensional figure corresponding to the heat map in Fig. 7 (right) is shown in Appendix A (Fig. 12).

## Discussion

This analysis is the fourth in a series of quantitative approaches to tumor growth data in which we present a new series of models capturing combination treatments. Figure 1 illustrates the progression of models and TSE concepts through these analyses. Models have been developed that describe chemical combinations as well as radiation combined with chemical intervention [15, 16]. Important biological features have been captured such as natural cell death, tumor regrowth, and eradication. This shows how modeling can improve our understanding of the target biology from a macro-perspective. TSE has evolved from a value [22], to a curve, or surface, to include radiation, and to describe long-term or irreversible effects. TSE_dV_ curves and heat maps provide a more nuanced understanding of tumor evolution, beyond the binary of tumor growth or shrinkage. These generalizations are important to capture as many treatment forms and effects as possible. TSE can also be used to support the selection of compounds in the discovery process (Cardilin et al. 2019, preprint).

### Exposure to radiosensitizer

Pharmacokinetics of the radiosensitizer was adequately described by a one-compartment model. The estimated half-life of 3 h gives no accumulation of drug with the current administration schedule. Although the radiosensitizer was given orally, absorption was not included in the final model. Data only allowed to develop a disposition model which was sufficient to describe the plasma concentration at the time of irradiation. This approach was conservative in the sense that it avoided underestimating the plasma concentration at the time of irradiated, which would have led to an overestimation of the radiosensitizer potency.

### Tumor model of radiation and radiosensitizer combination treatment

**Fig. 4:**
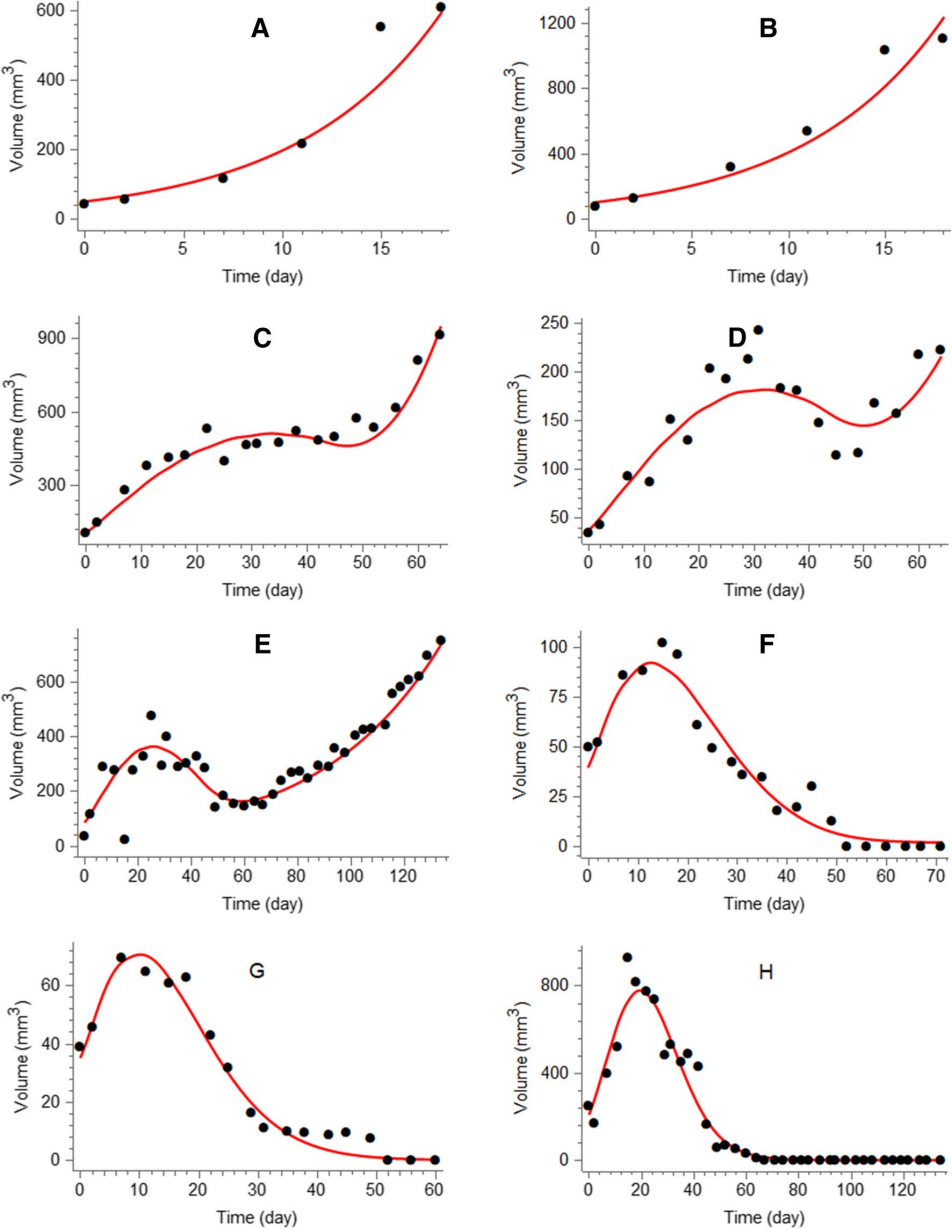
Experimental data (symbols) and model predictions (solid curves) of tumor volume versus time for the four treatment groups: **a**, **b** vehicle control, **c**, **d** radiation (2 Gy per dose), **e**, **f** combination treatment with radiation (2 Gy per dose) and radiosensitizer (25 mg kg^−1^ per dose), and **g**, **h** combination treatment with radiation (2 Gy per dose) and radiosensitizer (100 mg kg^−1^ per dose). Treatment was repeated 5 days a week for 6 weeks. Data are also shown in Appendix A (Fig. 8) using common volume and time scales

The proposed tumor model (Fig. 2) was able to simultaneously describe all four treatment groups. Vehicle growth was adequately explained by an exponential growth function (Fig. 4a, b). The estimated $k_{\text{g}}$ of 0.4 h^−1^ is similar to the earlier value of 0.5 h^−1^ for the same cell line [15]. In general, the net growth rate can range from 0.05 to 0.5 h^−1,^ which is comparable to our estimate $k_{\text{g}} - k_{\text{k}}$ = 0.14 h^−1^ [9, 16, 22–28]. The initial distribution of tumor volume was chosen to achieve a net growth rate $k_{\text{g}} - k_{\text{k}}$, which also meant that part of the initial volume was made up of nonproliferating cells. This leads to a larger estimated growth rate $k_{\text{g}}$ compared to if the initial volume was made up of only proliferating cells, and avoids under predicting the necessary exposure for tumor regression, i.e., TSE. Other commonly employed growth functions include Gompertz, (generalized) logistic, and Simeoni [8]. A Gompertz model was also fitted to the data, but the additional capacity parameter could not be reliably estimated. Radiation treatment (Fig. 4c, d) was sufficiently described by a combination of radiation-induced cell killing and growth inhibition. Cell killing by apoptosis (and possibly other death mechanisms) was also featured in a previous analysis [15]. The fraction of lethally irradiated cells was chosen according to the LQ model, an approach employed by Okumura et al. and recently by Tariq et al. [23, 29]. However, our inclusion of compartments $U_{i}$ to allow lethally irradiated cells additional cell divisions was a novelty [15]. This idea is supported by experiments, showing that irradiated cells can survive one or multiple cell cycles before dying through mitotic catastrophe [17, 18]. A similar idea was proposed by Watanabe et al. who modified the transfer between the proliferating and dying states to account for additional cell cycles [24]. However, that model only described response to a single dose of irradiation, not a combination with chemical intervention. Moreover, Watanabe et al. discussed the impact of radiation damage to the vasculature structure and the possibility of describing such an effect in their model [24]. In our model, the effect of radiation damage to the vasculature structure and tumor microenvironment was described as a permanent inhibition of the natural growth rate. This was needed to capture two types of tumor evolution that were clearly seen in the data (Fig. 4): complete tumor eradication, and tumor regrowth with different rates. Slower regrowth compared to control animals was clearly observed in our data and could be attributed to mutations and changes in the tumor microenvironment caused by irradiation [19]. In particular, radiation is known to reduce vascularization in the tumor and surrounding tissue, leading to hypoxia and reduced growth [30, 31]. It is noteworthy that both parameters related to radiation ($\alpha$ and $\gamma$) were estimated with high precision, emphasizing the need for both short- and long-term effects to describe the data. In contrast to the proposed model that features permanent inhibition of growth, other models have featured a repair process of DNA damage [13, 14]. This was not possible to do with our model due to data showing no signs of recovery in growth rate, which makes it similar to a model reported by Querdani et al. that employs a permanent inhibition of vascularization after administration of the drug Pazopanib to describe long-term treatment effects on tumor volume [25]. Unlike the radiation models mentioned above, the proposed model also accounts for combinations with radiosensitizing treatments. This is described generically as an enhancement of the radiation effects. The presence of a radiosensitizer will, therefore, lead to the same tumor evolution as if a higher dose of radiation had been administered. The radiosensitizer parameters ($a$ and $b$) were estimated with worse precision than the other parameters. This is likely due to the large variability in radiation effects, which makes it more difficult to quantify the differences between radiation and combination treatments.

### Tumor static exposure

Tumor static exposure is an important concept that can be used for single-agent treatment as well as combination therapies [15, 16, 20, 26, 27]. Historically, TSE values and TSE curves are used to predict the required exposures for tumor regression, although they have also been used for in vitro–in vivo correlations [31]. The first TSE concept described net growth rate at an arbitrary time point [16, 20]. This means that in order for the tumor to shrink, concentrations need to be maintained above the TSE curve for a prolonged period of time. Another type of TSE curve featured a daily perspective [15]. The radiation dose is the daily radiation dose and the plasma concentration is the daily average, and if exposure above the TSE curve is maintained over many days, the tumor will shrink. Finally, the TSE curve presented in this analysis (Eq. 11) examines permanent inhibition of growth. The radiation dose is the total radiation dose and radiosensitizer concentration is the concurrent plasma concentration at each instance of irradiation. All three TSE concepts describe treatments with one adjustable feature (the minimum, average, or total exposure) with the common objective of achieving tumor regression while putting low metabolic pressure on the animal.

**Fig. 5:**
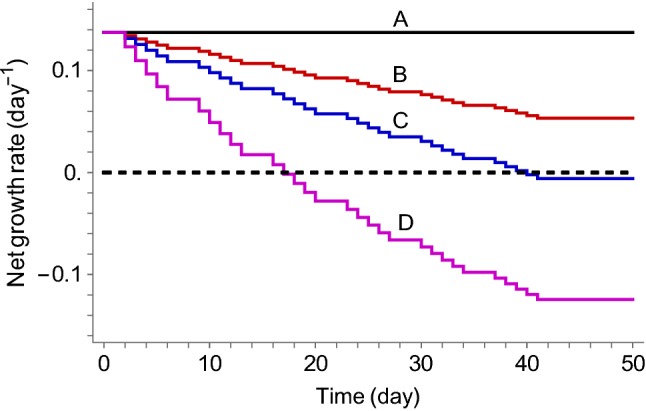
Evolution of net growth rate ($k_{\text{net}}$) over time due to radiochemical inhibition of growth for the following four treatment groups: (A) vehicle, (B) radiation treatment (2 Gy per dose), (C) radiation (2 Gy per dose) and radiosensitizer (25 mg kg^−1^ per dose) combination treatment, and (D) radiation (2 Gy per dose) and radiosensitizer (100 mg kg^−1^ per dose) combination treatment

Figure 5 shows how the net growth dynamically changes over time depending on treatment. The figure serves as a precursor to TSE, since it is based on the same expression, i.e., the net growth rate (Eq. 9). The projected changes in growth rate for the different treatment groups (A–D) show whether or not the corresponding dosing schedule will result in exposure above TSE (i.e., tumor shrinkage) or below TSE (i.e., tumor growth). In Fig. 5, this means that vehicle and radiation treatment must fall below the TSE curve, whereas combination treatment with the radiosensitizer results in a negative net growth rate and must, therefore, correspond to exposures above the TSE curve. Moreover, Fig. 5 shows the predicted net growth rates beyond simply whether or not the rate will be negative, which is similar to the heat map in Fig. 7 (right).

**Fig. 6:**
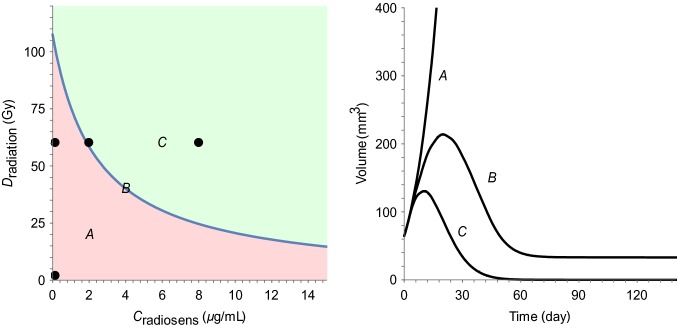
(Left) The TSE curve for radiation and radiosensitizer combinations is shown in blue. Exposure pairs (total radiation doses and concurrent plasma concentrations) above the curve (green area) will result in tumor regression and eradication, whereas exposure pairs below the curve (red area) are insufficient for regression and the tumor will continue to grow. (Right) Simulated tumor growth given three different exposure pairs (A, B, and C) marked in the TSE plot: A leads to tumor growth, B to tumor stasis, and C to tumor shrinkage

**Fig. 7:**
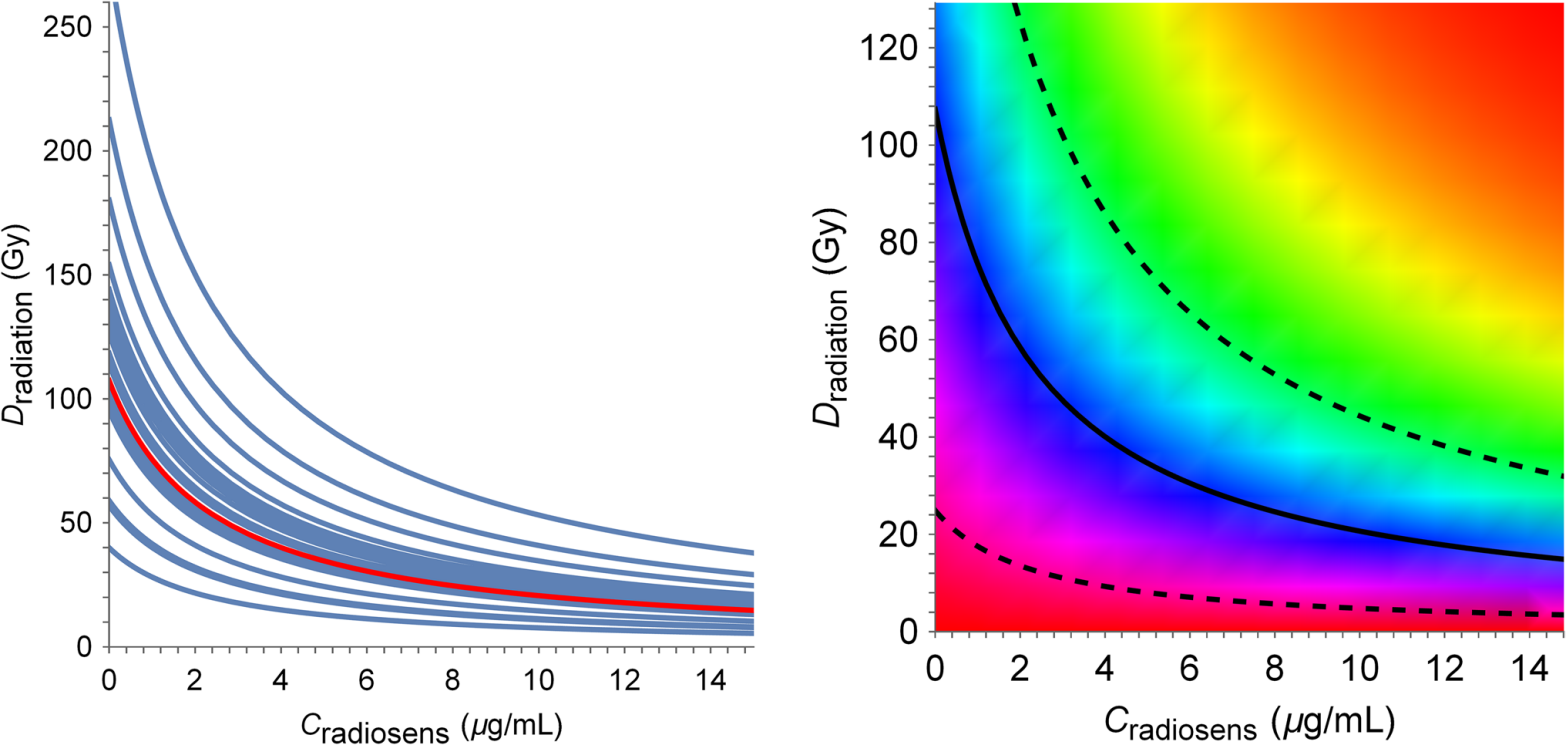
(Left) Variability in TSE among the population. Each curve (blue) corresponds to the TSE for a specific individual, based on the EBEs obtained from mixed-effects modeling. The median TSE curve is shown in red. (Right) Heat map of the net tumor growth rate after combination treatment with radiation and radiosensitizer for the median individual. The TSE curve from Fig. 5 is shown in black. Dashed lines indicate growth rates of 0.1 h^−1^ (corresponding to a doubling time of about 7 days) and − 0.1 h^−1^ (corresponding to a half time of about 3 days), respectively

**Fig. 8:**
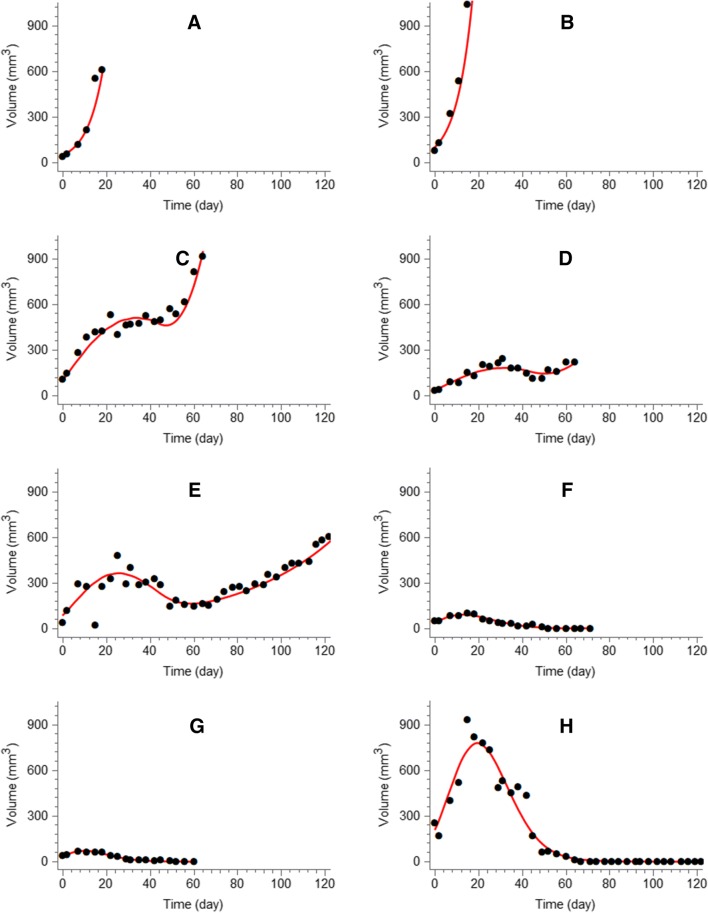
Examples of time courses of tumor evolution and model predictions based on the model in Eq. 5 and the parameters from Table 1: **a**, **b** vehicle, **c**, **d** radiation (2 Gy per dose), **e**, **f** combination treatment with radiation (2 Gy per dose) and radiosensitizer (25 mg kg^−1^ per dose), and **g**, **h**: combination treatment with radiation (2 Gy per dose) and radiosensitizer (100 mg kg^−1^ per dose). Treatment was repeated 5 days a week for 6 weeks. The figure is a version of Fig. 3 with the same time and volume scales for all individuals

**Fig. 9:**
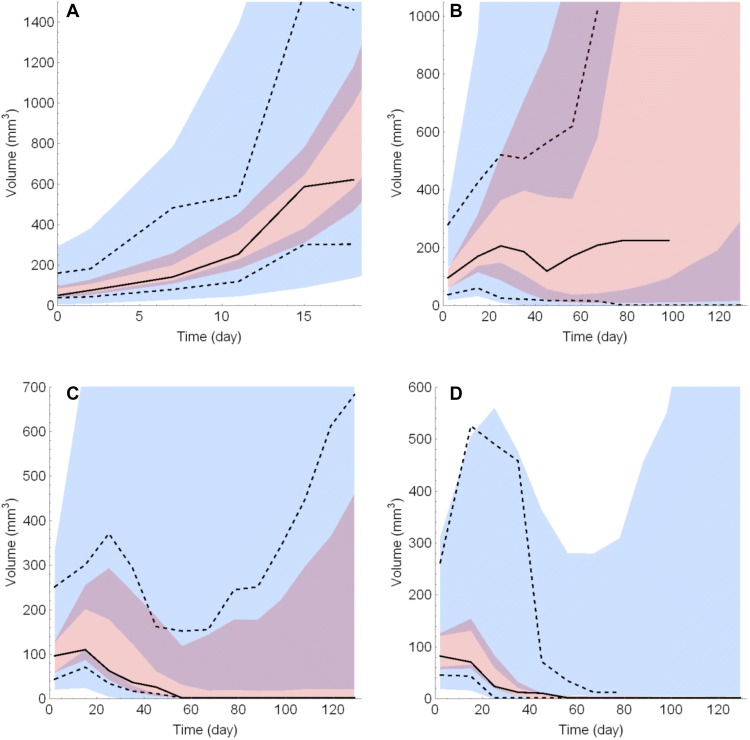
Visual predictive checks for each of the four treatment groups: **a** vehicle, **b** radiation treatment with 2 Gy per dose, **c** combination treatment with 2 Gy radiation and 25 mg kg^−1^ radiosensitizer per dose, and **d** combination treatment with 2 Gy radiation and 100 mg kg^−1^ radiosensitizer per dose. Shaded regions indicate 90% confidence intervals obtained by simulating 1000 studies according to the model (Eq. 5) and the parameter estimates in Table 1, for the 10th, 50th (median), and 90th percentiles. Observed medians are drawn in black solid lines, and observed 10th and 90th percentiles in black, dashed lines

**Fig. 10:**
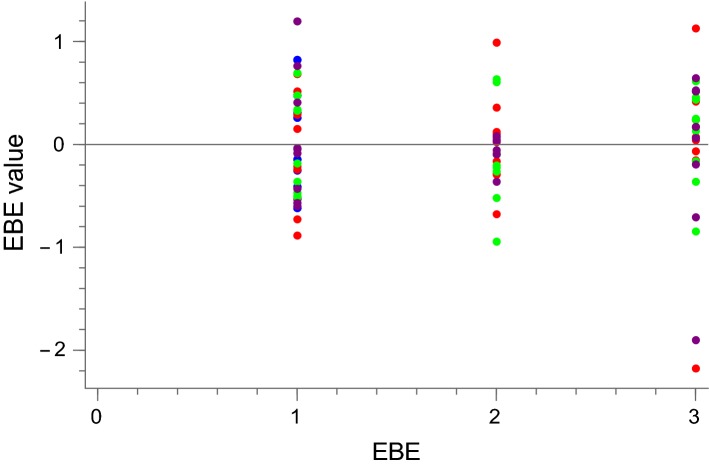
EBEs for each of the parameters $V_{0} , \;\gamma$, and $\alpha$ (1, 2, and 3, respectively) for each of the four treatment groups: vehicle (blue), radiation (red), radiation and 25 mg kg^−1^ radiosensitizer (green), and radiation and 100 mg kg^−1^ radiosensitizer (purple). Shrinkage can be seen in the parameter $\gamma$ (2) for the last group (purple) due to all tumors being eradicated in that group

The TSE curve in Fig. 6 has a pronounced curvature, which shows that the total radiation dose can be significantly decreased if combined with radiosensitizing treatment. When radiation is given alone, the TSE prediction is that 110 Gy is needed to eradicate the tumor. This is consistent with experiments using the same cell line, which puts the required dose between 100 and 120 Gy [32, 33]. The TSE curve in Fig. 6 is based on the median individual, which means that, in a heterogeneous population, approximately half of the individuals will need exposures above TSE to achieve tumor regression. This is why mixed-effects modeling, which quantifies between-subject variability, is important. The individual TSE curves (Fig. 7, left) make it possible to target tumor regression for a large percentage of the population, e.g., Fig. 7 predicts that for radiation treatment alone to achieve tumor eradication in the majority of the population, the required dose would be around 160 Gy. The heat map (Fig. 7, right) can be seen as a generalization of the TSE concept. TSE divides exposure into tumor growth and tumor shrinkage, whereas the heat map shows the resulting growth rate or shrinkage rate for different levels of exposure. In particular, TSE_dV_ curves corresponding to different growth rates need not have the same curvatures and the degree of synergy may, therefore, vary for different shrinkage rates (Fig. 7 black, dashed). This is consistent with the idea that drug synergies can be exposure-dependent [6].

### Applications and translation to humans

The proposed model can be used for radiation in combination with a range of chemical interventions. This includes immunotherapy, which is becoming increasingly important in oncology [34], as well as compounds that target DNA repair and replication stress [35]. The model is relatively simple and can be calibrated using data from the standard xenograft experiments. The model and the TSE concept have multiple applications. A typical application is to generate treatment predictions for different dosing schedules. The TSE curve itself is also a prediction and attempts to answer the question “how much exposure is needed for tumor shrinkage?”. The TSE curve can be used to determine drug synergies, which are related to the curvature, with synergy resulting in an inward curvature, whereas antagonism gives an outward curvature [16]. TSE can also be used as a basis for comparing and ranking compounds and combinations during drug discovery (Cardilin et al. 2019, preprint). Finally, the model and TSE have translational potential [36].

**Fig. 11:**
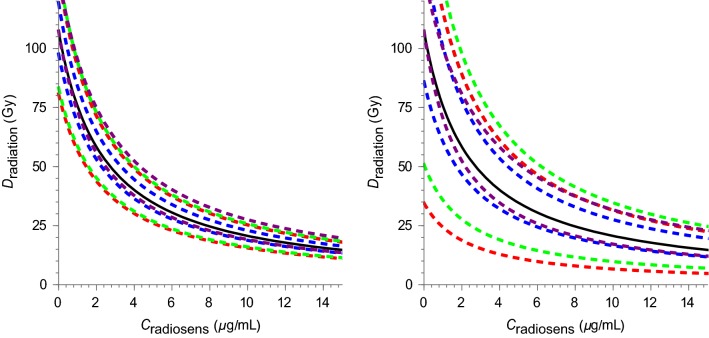
Sensitivity analysis of TSE curve. One at a time, each of the four model parameters $k_{\text{g}}$ (red), $k_{\text{k}}$ (green), $\gamma$ (blue), and $b$ (purple) was increased or decreased by 10% (left) or 25% (right). Largest changes in TSE were observed when the system parameters $k_{\text{g}}$ or $k_{\text{k}}$ were adjusted

**Fig. 12:**
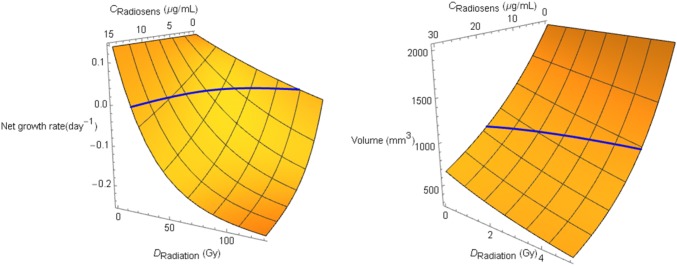
(Left) Simulated response surface with respect to net growth rate ($k_{\text{net}}$) given by Eq. 7. The TSE curve (Fig. 5, left) which corresponds to zero net growth is shown in blue. (Right) Simulated response surface with respect to tumor volume after 25 days. Combinations of radiation and radiosensitizer that result in 50% reduction of tumor volume compared to vehicle are shown in blue

Translational models are an important tool in drug development, but must account for species differences in pharmacokinetics as well as pharmacodynamics [6]. If there are no available data on human pharmacokinetics, a standard approach is to employ allometric scaling [37]. Wong et al. compared the treatment response in subcutaneous mouse models with the clinical response and found a correlation only when the quantitative tumor models were driven by human pharmacokinetics [38]. Mager et al. note that turnover rates are typically allometrically scalable, whereas capacity and sensitivity parameters often remain the same across species [39]. However, higher energy turnover in smaller animals to maintain 37 °C may affect target turnover and, therefore, also in vivo potency [40]. Moreover, hyper-inflammation cancerous states affect protein synthesis and degradation, and can, therefore, also impact potency [41]. Gabrielsson et al. have pointed out how differences in drug-target binding, target turnover, and drug partitioning can help explain differences across species [42]. An allometric scaling approach showed that if only the system rate parameters ($k_{\text{g}}$ and $k_{\text{k}}$) are scaled, the TSE curve does not change, although the tumor growth trajectories are affected [36]. More precisely, TSE only depends on the quotient $k_{\text{g}} /k_{\text{k}}$, which does not change if $k_{\text{g}}$ and $k_{\text{k}}$ are both scaled. However, the difference $k_{\text{g}} - k_{\text{k}}$ will be affected by scaling and the tumor will, therefore, grow slower in a larger animal (e.g., a human). The situation becomes considerably more complicated if drug and radiation parameters are also expected to vary across species and can lead to TSE curves corresponding to much greater exposure levels and different curvatures/drug synergy. A first step could be to investigate the sensitivity of the TSE curve to changes in drug/radiation parameters (see Appendix A, Fig. 11).

## Conclusions

The tumor model for treatment with radiation and radiosensitizing agents that we present can describe long-term treatment effects including tumor regrowth and tumor eradication. The model can be calibrated using tumor volume data obtained from standard xenograft studies. The TSE concept is extended to determine combinations of radiation dose and radiosensitizer concentrations that lead to tumor eradication. TSE is also extended by means of a heat map that provides information about the rate at which tumor growth or tumor regression is occurring.

To further establish applicability, the tumor model, as well as the TSE concept and heat map, should be challenged by data from different studies, using different types of radiosensitizers and different radiation doses. It is also important to test how the model translates to the clinic, e.g., how well the predicted TSE curves hold in a clinical setting.

## Appendix A

See Figs. 8, 9, 10, 11, and 12.
